# Supplementary figures and images for: The ancient and helical architecture of Elasmobranchii’s spermatozoa enables progressive motility in viscous environments
Source: PLoS One. 2025 Feb 25;20(2):e0319354. doi: 10.1371/journal.pone.0319354 (PMC11856307; doi:10.1371/journal.pone.0319354)

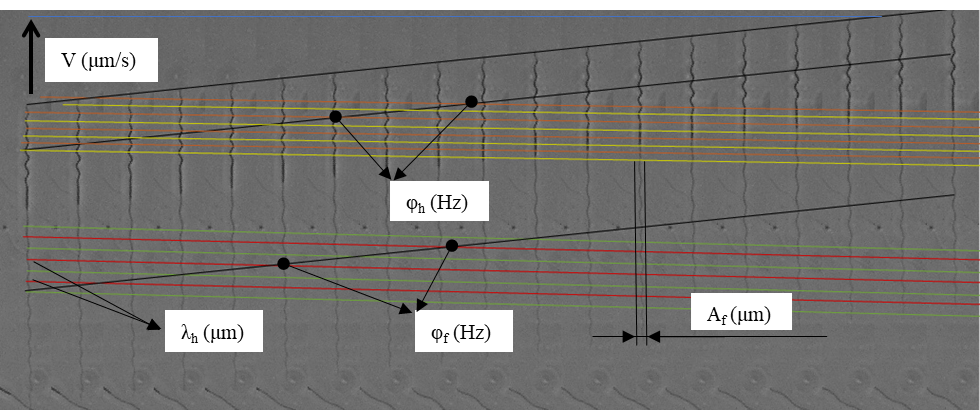

Supplement: S1 Fig — The black line corresponds to the line connecting the tips of the head on each frame. The yellow and orange lines connect the corresponding “waves” on the spermatozoa head, and the green and red connect waves on the tails. By calculating intervals (number of frames) of the line of the same colour crossing the black line, we can estimate the time needed for one complete rotation or beat cycle (0.05 s between each frame). The number of such cycles per second will be expressed as frequency (Hz). The distance between the green and red lines provides information about the length of the flagellar wave (μm). Recalculating the distance spermatozoa tip travelled during one second will give us info about sperm velocity V (μm/s). The amplitude of the flagella wave is the distance between two parallel lines connecting the waves of the flagellum from both sides, A (μm). Related to the Methods section. (TIF) [file pone.0319354.s002.tif]

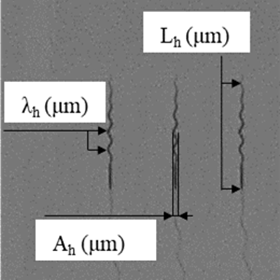

Supplement: S2 Fig — The distance from the tip of the head to the beginning of the flagellar is measured as head length (including midpiece). The distance between the two parallel lines connecting the waves of the head helix from both sides is the amplitude of the head helix. The length of the head helix is measured as the average distance between two wave crests on one side of the head. The number of helices is calculated as the total number of all crests on both sides of the head divided by 2. Related to the Methods section. (TIF) [file pone.0319354.s003.tif]
