# Supplementary material for: The ancient and helical architecture of Elasmobranchii’s spermatozoa enables progressive motility in viscous environments
Source: PLoS One. 2025 Feb 25;20(2):e0319354. doi: 10.1371/journal.pone.0319354 (PMC11856307; doi:10.1371/journal.pone.0319354)
Supplement: Attachment — Submitted filename: Response to Reviewers.docx [file pone.0319354.s008.docx]

*Dear Editor and Reviewer,*

*We would like to express our sincere gratitude to the expert reviewers for their specialist time, for the thorough examination of our paper, and for the detailed feedback provided. We feel that the manuscript has greatly improved after the insightful and constructive comments by the referee.*

*Please find below our point-by-point responses to each individual comment (highlighted in italics and blue colour).*

Reviewers'comments:

Reviewer's Responses to Questions

**Comments to the Author**

1. Is the manuscript technically sound, and do the data support the conclusions?

The manuscript must describe a technically sound piece of scientific research with data that supports the conclusions. Experiments must have been conducted rigorously, with appropriate controls, replication, and sample sizes. The conclusions must be drawn appropriately based on the data presented.

Reviewer #1: Yes

2. Has the statistical analysis been performed appropriately and rigorously?

Reviewer #1: N/A

3. Have the authors made all data underlying the findings in their manuscript fully available?

The [PLOS Data policy](https://eur02.safelinks.protection.outlook.com/?url=http%3A%2F%2Ftrack.editorialmanager.com%2FCL0%2Fhttp%3A%252F%252Fwww.plosone.org%252Fstatic%252Fpolicies.action%2523sharing%2F1%2F010f01942b4ab988-3e15776a-1bdb-42b8-8e72-275524ed8af6-000000%2FYT2zmZPPcYsGGhfeXujzHmZBPdn0kKUSNXWbJp342Ng%3D192&data=05%7C02%7Csboryshpolets%40frov.jcu.cz%7C26a0b1405db84697154708dd2bd10fa7%7Cc35f5da49a0344e68bf992833634f6a7%7C0%7C0%7C638714899538009353%7CUnknown%7CTWFpbGZsb3d8eyJFbXB0eU1hcGkiOnRydWUsIlYiOiIwLjAuMDAwMCIsIlAiOiJXaW4zMiIsIkFOIjoiTWFpbCIsIldUIjoyfQ%3D%3D%7C0%7C%7C%7C&sdata=JURsOyBxgM58ZH5xqYBInpAANfA4mavrKAvhbT27NoY%3D&reserved=0) requires authors to make all data underlying the findings described in their manuscript fully available without restriction, with rare exception (please refer to the Data Availability Statement in the manuscript PDF file). The data should be provided as part of the manuscript or its supporting information, or deposited to a public repository. For example, in addition to summary statistics, the data points behind means, medians and variance measures should be available. If there are restrictions on publicly sharing data—e.g. participant privacy or use of data from a third party—those must be specified.

Reviewer #1: Yes

4. Is the manuscript presented in an intelligible fashion and written in standard English?

PLOS ONE does not copyedit accepted manuscripts, so the language in submitted articles must be clear, correct, and unambiguous. Any typographical or grammatical errors should be corrected at revision, so please note any specific errors here.

Reviewer #1: Yes

5. Review Comments to the Author

Please use the space provided to explain your answers to the questions above. You may also include additional comments for the author, including concerns about dual publication, research ethics, or publication ethics. (Please upload your review as an attachment if it exceeds 20,000 characters)

Reviewer #1: This is an excellent paper highlighting sperm motility in three cartilaginous fish species. The authors have described sperm motility and sperm structure both qualitatively as well as quantitatively. They have comprehensively shown the importance of investigating sperm motility at high viscosities, presumably mimicking parts of the female reproductive tract where fertilization is likely to occur. While I believe they have performed a novel study of great interest I think they have underplayed the osmotic effects at least of seawater initiating further motility and hardly mention the really extremely important fact that sharks represent the only vertebrates having a blood osmotic concentration of about 1000mOsm/Kg or equivalent to seawater. More attention should have been devoted both experimentally as well as in the discussion on the flushing of sperm using the siphon organ to flush sperm along the clasper groves into the female reproductive tract. The authors should further explain the already activated sperm that are further activated and pushed towards the more viscous female environment. So, the authors should at least expand their discussion to include this.

*We sincerely thank Prof. Gerhard van der Horst for his thorough review of our manuscript and his valuable comments, as well as for his enthusiasm for this research. Below, we have provided detailed responses to each individual point.*

*We have now amended the text in the discussion section to reflect your suggestion, including more details:* "*All Elasmobranchii species are internal fertilizers. At the same time, the fertilization process itself is not fully understood in these species. Males deposit spermatozoa through specific grooves on claspers (extended pelvic fins) during mating [2]. However, these channels are not fully closed, allowing contact with the aquatic environment (seawater in marine species and freshwater in freshwater species). Also, the sperm is propelled into the female by the accumulation and subsequent release of water from the siphonal sacs in sharks [37, 38] or, presumably, by secretions from claspers glands in rays and skates [39]. Thus, sperm motility could potentially be activated by external factors similar to external fertilizers [40]. On the other hand, the unique physiology of marine Elasmobranchii species sets them apart from other vertebrates since their blood and seminal plasma osmolality is approximately 1000 mOsm/kg (equivalent to seawater), thus preventing osmotic shock during transition. In contrast, freshwater Elasmobranchii have internal body fluid osmolality ranging between 243–350 mOsm/kg. In this case, contact of spermatozoa with freshwater induces osmotic shock, potentially resembling signalling in freshwater external fertilizers. However, in our previous observations, freshwater led to a rapid cessation of flagellar beating [41]. These findings suggest that the motility of Elasmobranchii spermatozoa is not directly regulated by environmental osmolality. Nevertheless, further research is needed to explore the potential effects of the siphonal sac or clasper gland fluids on sperm physiology during mating and to uncover the precise mechanisms involved in sperm deposition and interaction with the female's internal environment. Our observations indicate that the spermatozoa flagellum remained actively beating in undiluted seminal fluid and ASF across all three species, even when spermatozoa were still compacted within the spermatozeugmata. When LVM was used, the spermatozoa were nonprogressive, tumbling in place, and struggled to leave the spermatozeugmata unless it was mechanically disrupted during mixing with ASF. Interestingly, an increase in ASF viscosity was sufficient to trigger progressive sperm motility. Thus, we suggested that during mating, the already activated spermatozoa (still packed in spermatozeugmata) are transported towards the female's internal organs, where the next phase of progressive motility is initiated upon encountering the more viscous internal environment."*

My other main criticism is that they have measured straight line length of sperm but not the true length. There are many simple image analysis programmes to accurately measure sperm head length following the helical sperm head length.

*Many thanks for this. Initially, we intended to measure the true length of sperm heads using image analysis software. However, we found that the 3D nature of the head and its irregular shape—varying helix pitch along the head—made these measurements biased and inaccurate. Thus, obtaining exact measurements that reflect the true length is challenging and would not influence the study's main findings. Instead, a straightforward approximation allows us to compare our data with previously published results, where straight-line length measurements were used. For example, see Tanaka S, Kurokawa H, Masako H. Comparative Morphology of the Sperm in Chondrichthyan Fishes. In: Jamieson B, G, Ausio J, Jean-Lou J, editors. Advances in Spermatozoal Phylogeny and Taxonomy. Muséum national d'Histoire naturelle, Paris: Editions du Muséum national d'Histoire naturelle; 1995. p. 313-20.*

My other comments are mainly minor and relate to grammar and typography. While I will list some examples here they need to get someone who is in command of really good scientific English to edit the paper thoroughly for scientific English

Some examples will suffice

*We are very grateful for pointing out this important omission in our manuscript. We have now endeavoured to remove typographical and grammar errors by thoroughly proofreading. Additionally, during manuscript preparation, the scientific English was thoroughly reviewed multiple times by us and our colleagues, and we believe it meets the standards of the journal. However, if the reviewer or editors feel it is necessary, we are open to submitting the manuscript for an additional professional English proofreading service.*

Line 39: low viscosity and real osmotic concentration

*The text of the abstract was corrected, and missing information was added*

Line 40: Even in abstract write out abbreviations such as MC

*The text was corrected, and abbreviations are described.*

Line 48: Modern not correct... should be currently or Surviving....

*The word "Modern" was replaced with "Currently living."*

Line 132: not possible... is not a good reason

*Replaced with "**challenging"*

Line 173: Write abbreviations in full when explaining/discussing for the first time

*The text was corrected, and the abbreviations are now described.*

Line 358: forces

*The text was corrected.*

Line 361: reference required

*The reference was added: 27.Andrietti F, Bernardini G. The movement of spermatozoa with helical head: theoretical analysis and experimental results. Biophysical Journal. 1994;67(4):1767-74. doi: 10.1016/S0006-3495(94)80651-4.*

Lines 397 to 400: This refers to my arguments above and the balance between just seawater activation, initial mixing with tubular fluid or experimental with methyl cellulose need to be better investigated/explained.

*Many thanks for this suggestion. The text in the discussion section has now been extended to include more details per your suggestion.*

Line 448: At least speculate on these aspects and maybe it is still the attachment/remnants of sheaths related to Sertoli cell "embedding material"

*Thank you for this suggestion. The text is corrected and extended: "The Hermes body, or the cytoplasmic droplet, is a component of the sperm flagellum unique to epididymal spermatozoa, originating as the remnant of germ cell cytoplasm during spermatogenesis. Its precise role and function are unclear, but several authors* *hypothesize that this structure might be associated with the maturation process and acquisition of sperm motility [56, 57].* *Alternatively, the observed sleeves could be reminiscent of the structural matrix that facilitates the interaction between Sertoli cells and developing spermatozoa during spermatogenesis since they are not a part of spermatozoa itself but rather just provide an additional layer or cover at the midpiece with this extra membrane."*

Finally, I like the discussing/hypothesizing around the issue of sperm shape/motility/penetration through mucous and potentially fertilization outcome. The videos are excellent and help to follow the arguments,

*We are incredibly thankful for your positive evaluation and enthusiastic feedback on our work.*

6. PLOS authors have the option to publish the peer review history of their article ([what does this mean?](https://eur02.safelinks.protection.outlook.com/?url=https%3A%2F%2Ftrack.editorialmanager.com%2FCL0%2Fhttps%3A%252F%252Fjournals.plos.org%252Fplosone%252Fs%252Feditorial-and-peer-review-process%2523loc-peer-review-history%2F1%2F010f01942b4ab988-3e15776a-1bdb-42b8-8e72-275524ed8af6-000000%2FJG_aRiaxNCxP_TgqcLiWZVpGTCOlTrqKNFZQpQRhpwg%3D192&data=05%7C02%7Csboryshpolets%40frov.jcu.cz%7C26a0b1405db84697154708dd2bd10fa7%7Cc35f5da49a0344e68bf992833634f6a7%7C0%7C0%7C638714899538023053%7CUnknown%7CTWFpbGZsb3d8eyJFbXB0eU1hcGkiOnRydWUsIlYiOiIwLjAuMDAwMCIsIlAiOiJXaW4zMiIsIkFOIjoiTWFpbCIsIldUIjoyfQ%3D%3D%7C0%7C%7C%7C&sdata=KfL%2FfUrpMch49Z%2B9KqUGESN42Y%2B7baKsrKLq3%2BN%2Bn1U%3D&reserved=0)). If published, this will include your full peer review and any attached files.

If you choose "no", your identity will remain anonymous but your review may still be made public.

**Do you want your identity to be public for this peer review?** For information about this choice, including consent withdrawal, please see our [Privacy Policy](https://eur02.safelinks.protection.outlook.com/?url=https%3A%2F%2Ftrack.editorialmanager.com%2FCL0%2Fhttps%3A%252F%252Fwww.plos.org%252Fprivacy-policy%2F1%2F010f01942b4ab988-3e15776a-1bdb-42b8-8e72-275524ed8af6-000000%2FtQv01CZqxCMOjuM2Q-lTqjyZcQeC8V8B4izlFE61dH8%3D192&data=05%7C02%7Csboryshpolets%40frov.jcu.cz%7C26a0b1405db84697154708dd2bd10fa7%7Cc35f5da49a0344e68bf992833634f6a7%7C0%7C0%7C638714899538036569%7CUnknown%7CTWFpbGZsb3d8eyJFbXB0eU1hcGkiOnRydWUsIlYiOiIwLjAuMDAwMCIsIlAiOiJXaW4zMiIsIkFOIjoiTWFpbCIsIldUIjoyfQ%3D%3D%7C0%7C%7C%7C&sdata=yEX4w8ypH5xcji5CF1so%2FIAsNwaM7gDsraKjm0zvTec%3D&reserved=0).

Reviewer #1: **Yes:** Prof Gerhard van der Horst
